# Domains of Capacity Building in Whole-Systems Approaches to Prevent Obesity—A “Systematized” Review

**DOI:** 10.3390/ijerph191710997

**Published:** 2022-09-02

**Authors:** Sisitha Jayasinghe, Robert Soward, Lisa Dalton, Timothy P. Holloway, Sandra Murray, Kira A. E. Patterson, Kiran D. K. Ahuja, Roger Hughes, Nuala M. Byrne, Andrew P. Hills

**Affiliations:** 1 College of Health and Medicine, University of Tasmania, Launceston, TAS 7250, Australia; 2 College of Arts, Law and Education, University of Tasmania, Launceston, TAS 7250, Australia; 3 School of Health Sciences, Swinburne University of Technology, Melbourne, VIC 3122, Australia

**Keywords:** whole-systems approach, prevention, capacity building, overweight and obesity, community intervention

## Abstract

Despite increased awareness of its risks, for the most part, contemporary efforts for obesity prevention have been patchy at best. As such, the burgeoning interest in whole-systems approaches (WSAs) that acknowledge the complex, dynamic nature of overweight and obesity and operate across multiple levels of society is particularly timely. Many components of “community capacity building” (CB), an essential but often neglected aspect of obesity prevention, overlap with “best practice principles” in effective/optimal community-based obesity-prevention initiatives. Rhetoric urging WSAs and community CB in public health abounds although operative and efficacious contemporary examples of these approaches to reducing obesity levels are scarce. The aim of this investigation was to undertake a systematized review of the level of capacity building incorporated in published literature on WSAs targeting obesity to better understand how domains of CB have been incorporated. A *PubMed* search and a recently published systematic review were utilized to identify WSAs to obesity prevention between 1995–2020. A team-based approach to qualitative thematic data analysis was used to systematically assess and describe each intervention regarding explicit capacity-building practice. Despite not being specifically designed for building capacity, a significant proportion of the WSAs studied in the current report had implemented several CB domains.

## 1. Introduction

Despite increased awareness of the risks of obesity, progress in prevention in the past 20 years has been patchy at best [1]. To place the scale of the obesity problem in context, it is estimated that around 2.8% of global GDP (~USD 2 trillion) is wasted on obesity and associated health complications [2]. As illustrated in comprehensive models such as Foresight [3], the multifarious and complex nature of obesity etiology is widely accepted [4]. Traditional interventionist approaches, predominantly based on the linear model of cause and effect, have preoccupied obesity-prevention efforts for much of the late 1990s and early 2000s, with many now being outdated or obsolete. Accordingly, there has been a burgeoning interest in whole-systems approaches (WSAs) that acknowledge the complex, dynamic nature of overweight and obesity and operate across multiple levels of society (individuals, communities, governments, health systems, etc.) [5]. 

Unfortunately, the sustainability and effectiveness of preventative interventions have commonly been hampered by their relatively narrow focus on delivering a “package” of activities and/or educational messages [6]. Well-planned, well-resourced and optimally implemented WSAs provide a logical alternative. Community engagement, program design/planning, evaluation, sustainable implementation, and governance are reported as “best practice principles” in effective/optimal community-based prevention initiatives [7]. Interestingly, many of these items are also components of “community capacity building”, an essential but often neglected aspect of obesity prevention. As is often the case in a range of areas, the concept of capacity building has been inconsistently defined, and this has resulted in the generation of a raft of definitions over the years [8,9]. Capacity building (CB) is an increasingly important strategy for communities to promote security, development, and sustainability. It encompasses a broad range of activities that aim to strengthen the ability of people to manage their own challenges and to achieve development objectives in the context of sustainability. CB assumes that local actors are best situated to understand and therefore address their own community challenges, and assisting them is more likely to lead to effective, legitimate, and sustainable outcomes. In this vein, CB, theorized as a problem-solving endeavor [10], is now recognized as an alternative to the ambitious and expensive liberal interventionism that was popular during the 1990s and 2000s [11]. CB is therefore emerging as an effective avenue for communities to take ownership of issues with an opportunity to use existing capabilities within individuals, organizations, and systems to make progress. The World Health Organization (WHO) has defined capacity building as “the development of knowledge, skills, commitment, structures, systems, and leadership to enable effective health promotion with actions to improve health at 3 levels: the advancement of knowledge and skills among practitioners; the expansion of support and infrastructure for health promotion in organizations, and; the development of cohesiveness and partnerships for health in communities” [12,13]. 

Rhetoric urging WSAs and community capacity building in public health abounds although operative and efficacious contemporary examples of these approaches to reducing obesity levels are scarce [4,14]. Numerous past examples related to public health emergencies of global significance (such as tobacco smoking, HIV, diabetes, etc.) have illustrated how WSAs can be effectively utilized to achieve positive health outcomes [15,16,17,18]. To the best of our knowledge, no previous research has specifically investigated CB within WSAs to prevent obesity. The aim of this investigation was to undertake a systematized review of the level of capacity building incorporated in published literature on WSAs targeting obesity to better understand how domains of CB have been incorporated.

## 2. Materials and Methods

### 2.1. Search Strategy

**Figure 1:**
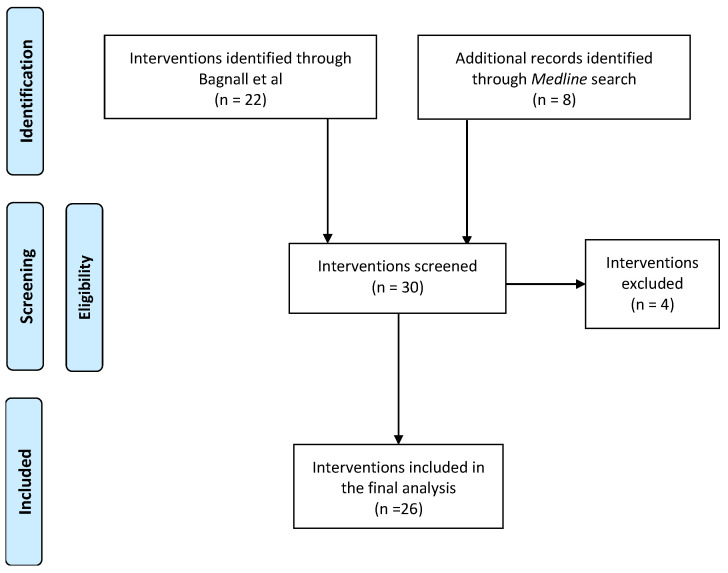
Summary of study selection.

Two main strategies were utilized to identify whole-system approaches (WSAs) to obesity prevention between 1995–2020. First, a recent systematic review by Bagnall and colleagues [5] was utilized to identify obesity-prevention-specific WSAs between 1995 and 2018. Bagnall and colleagues defined WSAs as those that (1) consider, in concert, the multifactorial drivers of overweight and obesity; (2) involve transformative coordinated action (including policies, strategies, and practices) across a broad range of disciplines and stakeholders, including partners outside traditional health sectors; (3) operate across all levels of governance, including the local level, so that such approaches are reinforced and sustained, and; (4) identify and target opportunities throughout the life course (from infancy to old age). A subsequent *Medline* database search was conducted (April 2020) to source information pertinent to other WSAs to obesity prevention from 2018–2020 (Figure 1). Key search terms included “obesity prevention” and “whole systems approach”, and these were used individually and in combination. All interventions were screened for appropriateness using the criteria listed in Bagnall et al. [5] and the National Institute for Health and Care Excellence (NICE) guidelines for WSAs [19]. Briefly, the existence of multiple “features”, including identification of a system, capacity building, creativity/innovation, relationships, engagement, communication, embedded action/policies, sustainability, leadership, and progress monitoring, was evaluated to ascertain eligibility. All studies that did not have a focus on obesity prevention or did not satisfy the WSA criteria were promptly excluded. As interventions were not screened based on study design, a wide array of experimental designs was incorporated in the final analysis. These included mixed-methods evaluations, randomized controlled trials, non-randomized controlled trials, qualitative or case studies, cross-sectional studies, natural experiments, and prospective cohort studies. 

### 2.2. Capacity Building Analysis

A team-based approach to qualitative thematic data analysis was used to manage the analytical workload. Each of the interventions was initially analyzed using an in-house capacity-building assessment model developed by one of the investigators (RH) [20]. A framework of 7 domains (leadership, intelligence, partnerships, workforce, community, project management, and resource mobilization) was utilized to systematically assess and describe each intervention regarding explicit capacity-building practice. The capacity domains were extracted from a Delphi study that investigated the content validity of capacity building conceptual framework originally proposed by Baillie and colleagues [21]. Focus questions were utilized to elicit pertinent information against the 7 CB domains (Table 1). Interventions were given a quality rating for each of the domains based on the level of evidence available (i.e., 1 = little evidence of explicit reference/strategy; 2 = some evidence; 3 = considerable evidence), and a final capacity-building score out of a maximum of 21 was derived. Subsequently, a descriptive thematic summary about capacity building was generated for each intervention. The CB analysis was performed by 3 independent investigators, and the final triangulated results are presented in this report. Majority consensus and stability consensus were both utilized [22] as a means of reaching agreement between investigators.

## 3. Results

**Figure 2:**
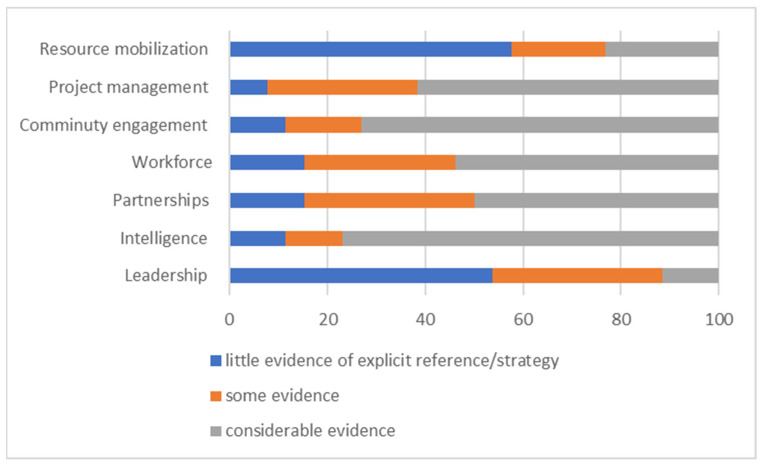
Levels of evidence pertaining to capacity building domains.

Twenty-seven instances of WSAs to obesity prevention were selected and included in the final analysis (Figure 1, Table 2). Three interventions that did not meet the inclusion criteria were promptly excluded. Selected interventions were from four geographical regions, with the majority based in the United States of America (Table 2). Most of these interventions focused on childhood obesity prevention, with a small number investigating maternal obesity and obesity associated chronic disease (Table 2). Similarly, most of the interventions (n = 15) were clustered at the top end (i.e., >17) of the capacity-building score spectrum—only 2 interventions scored ≤8. In most instances, there was considerable evidence on substantial community engagement and intelligence gathering, whereas explicit detail on leadership and resource mobilization were scarce (Figure 2). 

## 4. Conclusions

The present study assessed the extent to which components of CB are ingrained within the designs of key WSAs to obesity prevention from the recent past. A system usually contains many component parts that are in a perpetually dynamic interaction. Hence, the hallmark of a system is that none of its parts is completely independent [67]. To this end, practicability of WSAs to obesity prevention can be extremely complicated. Therefore, it is feasible neither to have an overarching working model of CB that will be unequivocally effective in a wide range of settings nor to expect various WSAs to have implemented the different domains of CB to the same extent. However, despite not being explicitly designed for building capacity, a significant proportion of the WSAs studied in the current report had implemented several CB domains, which is promising. This implies that obesity prevention and CB are not mutually exclusive and that WSA approaches must include as many domains of CB as possible in their design/implementation approaches. This also negates the typical notion that CB should be and is a subconscious, invisible practice [68]. The idea of a nuanced and/or comprehensive understanding of CB as a public health strategy should be entertained, and the routine incorporation of components of CB into public heath intervention design should be actively encouraged. 

The lack of explicit evidence of reference and/or strategy regarding leadership and resource management was of particular concern. “Leadership” is a vital component that plays a major role in several aspects of any WSA to obesity prevention, including adoption of the intervention, implementation, evaluation, and dissemination of the findings. Traditionally, leadership has been linked with influence and power and has often been associated with individuals, agencies, organizations that have the knowledge, control, and responsibility to change environments, policies, and practices related to obesity. However, if we draw on Foucault’s (1991) argument that “power is everywhere”, we can depart from the assumption that only particular actors use power as an instrument of coercion or influence and even away from the discreet structures in which those actors operate. Instead, we recognize that power is diffused and embodied in discourse and knowledge [69,70]. By doing so, we acknowledge that there is a considerable degree of variability in who and/or what can be considered a “leader” in the contemporary obesity-prevention landscape. To this end, current literature is replete with examples of the importance of “movers and shakers” of the community, i.e., “community champions”, in the efficacious implementation of WSAs to prevent obesity [71,72,73,74,75]. This indicates that a diverse range of formal and informal influencers and role models can fulfill the leadership that is required in community-based obesity prevention. As has been postulated in numerous theoretical examples, different types of leaders and leadership styles may be pertinent in a public health context. However, given the centrality of community participation and coalition formation in WSA approaches, it could be argued that a democratic and/or a laissez-faire type of leadership is most suitable for effective outcomes [76]. These leadership approaches lend themselves to facilitating capacity building from a bottom-up approach that draws on and strengthens existing local capacities by acknowledging all stakeholders as capable agents. 

Given the enormity of the challenge, the importance of resource mobilization in obesity prevention cannot be underestimated. Any efficacious attempt to curtail the current trends of obesity will undoubtedly require a vast amount of human, financial, and other resources. As such, it is perplexing that specific strategies for resource mobilization and management have not been included in many of the WSAs considered. It is also noteworthy that effective resource management can also have a significant impact on the sustainability of obesity-prevention efforts [77]. For instance, there is substantial evidence that availability of finances and effective management of such resources can single-handedly determine the longevity and scalability of most obesity-prevention efforts [78]. Accordingly, there is increasing interest by contemporary interventionists regarding novel, cost-effective strategies in implementing WSAs to obesity prevention. To this end, asset-based community development approaches, which consider sustainability as a core aspect and actively seek out opportunities to link extant micro-assets to the macro-environment, are a step in the right direction. 

The “systematized” and convenient nature of the study selection approach implemented in the current study could have potentially introduced bias and human error, which may have resulted in some relevant studies being missed. Nevertheless, the approach undertaken was a pragmatic necessity. It also noteworthy that the some of the included studies did not entail CB as one of their primary aims, which may have affected the level of overall identifiable CB elements. Further, the generalizability of the findings may be limited given the lack of WSAs from some geographical areas. It is well-known that obesity prevalence displays heterogeneous epidemiological patterns that are not readily explained in divergent geographical settings [79]. Future research may also benefit from investigating whether there is a dose–response relationship between domains of CB implemented and positive research outcomes. 

## Figures and Tables

**Table 1:** Focus questions for the domains of capacity building.

Capacity Domain	Focus Questions Evidence of:
**Leadership**	Reference to leadership (political, organizational, community, workforce)? Leadership development strategy?
**Intelligence**	Target population involved in identifying issues/solutions? Needs assessment conducted? Problem analysis clear? Specific analysis to inform intervention design? Evaluability assessment? Intervention evaluation?
**Partnerships**	Partnerships to address the issue? Strategies to build partnerships? Evaluation of partnerships?
**Workforce**	Specific workforce identification? Workforce development strategy? (training, CPD, upskilling?)
**Community**	Community involved in needs identification? Community involved in strategy decision making? Community investing resources to address issue? Community development strategies explicitly described?
**Project Management**	Stakeholders engaged in intervention management and decision making? Intervention goals and objectives coherent (SMART) Implementation monitoring?
**Resource Mobilization**	Specific attempts to mobilize resources external to immediate project funding? Types of resources mobilized? Size of intervention investment (estimate: small (USD 50K), medium (<USD 250,000), large (>USD 500,000)

*The capacity domains utilized in the current instance were extracted from a Delphi study [20] that investigated the content validity of a capacity-building conceptual framework originally proposed by Baillie and colleagues [21]. The potential influence of these capacity domains on intervention outcomes may vary depending on the context. As with many other “complex concepts/systems” in public health, capacity building can contain many heterogeneous and fleeting elements; an emergent collective effect that is different from the any influence of the original components by themselves; and components that are subject to changing circumstances.*

**Table 2:** Capacity-building interventions.

Study Region	Study Description/Summary of Capacity-Building Evidence	Leadership	Intelligence	Partnerships	Workforce	Community	Project Management	Resource Mobilization	Capacity-Building Score
Australia	**Romp & Chomp:** The intervention activities had a strong focus on community capacity building and developing sustainable changes in areas of policy, sociocultural, and physical environments by using a socioecological framework. The Romp & Chomp action plan was developed with extensive community consultation and stakeholder engagement, and a management committee of stakeholders oversaw its implementation [23,24,25,26].	1	3	2	3	3	3	2	17
	**Be Active Eat Well:** Building capacity was one of the key foci of this intervention (Objective 1 out of 10). It included broad actions around governance, partnerships, coordination, training, and resource allocation [27,28,29].	2	3	2	3	3	3	3	19
	**Campbelltown—Changing Our Future:** The five-step approach: (1) set up a childhood obesity-monitoring system by collecting baseline data from children in primary schools across Campbelltown LGA to give a local context to the community when developing the systems map; (2) key stakeholders develop systems maps that inform the development of the interventions; (3) key stakeholders and community groups identify priority areas for action and form working groups; (4) implementation of the interventions; (5) evaluation of the interventions, entailing several important domains of CB [30].	1	3	1	1	3	3	1	13
	**Sustainable Eating Activity Change Portland:** This intervention utilized asset-based community development (ABCD)—a strategy promoting sustainable community development—alongside applications of a collective impact framework that focused on efforts to connect and mobilize the community to act [31].	1	3	1	1	3	3	1	13
	**WHO Stops Childhood Obesity:** Intensive training and support within each community was oriented around strengthening WHO systems building blocks (e.g., workforce development, resources intelligence) and the New South Wales capacity-building framework (e.g., partners and networks) in community settings. A key focus included mapping existing systems and using these maps to develop and implement whole-systems change with community members and implementation support to optimize interventions [32].	2	3	3	2	3	3	1	17
	**It’s Your Move—ACT:** Capacity building among school project officers and student ambassadors (workshop and training opportunities) was a primary goal. The Analyses Grid for Environments Linked to Obesity (ANGELO) framework was modified to incorporate the World Health Organization systems building blocks, which include leadership, information, financing/resources, partnerships, and workforce development, into the development and implementation of the project to reduce unhealthy weight gain among adolescents through comprehensive school- and community-based systems change [23,33,34,35].	2	3	3	3	3	3	3	20
United States	**Healthy Living Cambridge Kids:** A community-based participatory research approach (i.e., The Healthy Children Task Force (Task Force) was utilized to engage community members in all aspects of the intervention process from research questions to design/implementation of the study and analysis/dissemination of findings [36].	1	3	2	2	3	2	2	15
	**Childhood Obesity Prevention Demonstration Project:** This included an implementation of numerous multi-level, multi-setting interventions for preventing and reducing obesity among children in a community, which for the most part ran as a collaboration between government- and community-level stakeholders. A specific focus included the application of lessons learned (regarding obesity prevention) from other geographical regions [37].	1	1	3	3	1	2	3	14
	**The San Diego Healthy Weight Collaborative:** Jointly implemented strategies in a Latino, underserved community included: (1) building an effective and sustainable collaborative team; (2) disseminating a healthy weight message across sectors; (3) assessing weight status and healthy weight plans in primary care, school, and early childhood settings; and (4) implementing policy changes to support healthy eating and physical activity [38].	3	2	3	3	3	2	2	18
	**Shape Up Somerville:** A community-based participatory research approach was implemented that focused on facilitating collaborative partnerships with the communities in all phases of the research: identifying the problem; designing, implementing, and evaluating the intervention; and identifying how data would inform actions to improve health within the community. Community engagement consisted of several forms, including meetings, focus groups, and key informant interviews, and led to the formation of several Shape Up Summerville advisory councils that remained actively involved throughout the study [39].	1	3	3	2	3	3	1	16
	**Healthy Start Partnership:** Efforts were made to better understand the challenges faced by public health professionals in implementing environmental and policy interventions related to public health through (1) participant observation of regional- and county-level meetings and conference calls; (2) qualitative interviews with HSP partners; and (3) self-administered structured questionnaires with HSP partners [40].	1	2	3	2	2	2	1	13
	**Healthy Eating and Exercising to Reduce Diabetes:** A community-based participatory intervention was implemented to identify facilitators of and barriers to sustained community efforts to address social factors that contribute to diabetes (and health). A concerted effort was put in to identify proximate (e.g., knowledge, diet) and intermediate (e.g., access to fresh produce) factors that contributed existing health trends. Education and community training sessions were conducted through the mediation of the steering committee. Further, partnership building and sourcing additional funds was actively addressed throughout the lifetime of the study [41].	2	3	2	3	2	2	2	16
	**Healthy Eating, Active Communities Program:** Changes in foods and beverages sold at schools and in neighborhoods; changes in school and after-school physical activity programming and equipment; individual-level changes in children’s attitudes and behaviors related to food and physical activity; and HEAC-related awareness and engagement on the part of community members, stakeholders, and policymakers were achieved through: (1) Engaging parents and families as advocates for healthier food and physical activity; (2) developing policy advocacy capacity in residents; (3) committing healthcare spokespersons to testifying at school board meetings, planning commission meetings, and city council meetings, and (4) educating parents on how some businesses market unhealthy food and physical activity to children [42].	2	3	2	3	3	3	2	18
	**Baltimore Healthy Communities for Kids:** A mixture of policy working groups, systems science modelling, regular meetings with key stakeholder groups, trainings (in person and online) of food source owners and youth leaders, and social media campaigns were utilized to increase affordability, availability, purchase, and consumption of healthy foods by low-income African American children and reduce obesity [43].	3	3	2	3	3	3	1	18
	**Shape Up Under 5:** Stakeholder-driven community diffusion—a novel conceptual framework which entails many domains of capacity building—was implemented to better understand how and why stakeholder groups succeed and the conditions under which they create community-wide change in the context of childhood obesity [44].	1	3	2	2	2	2	1	13
	**Central California Regional Obesity Prevention Program:** Promoting of safe places for physical activity, increased access to fresh fruits and vegetables, and supporting the community and youth engagement in local and regional efforts to change nutrition and physical activity environments for obesity prevention was undertaken using a regionally localized/focused workforce development, community engagement, and policy change approach [45].	2	3	3	3	3	3	1	18
	**California Healthy Cities and Communities:** Numerous new health and well-being programs were developed, organizational policies and practices adopted, and new financial resources leveraged across 20 participating sites through organizational development (city governments, lead agencies and community organizations), enhancing leadership skills among community residents, strengthening relationships among neighbors, and providing opportunities for residents to get involved in the civic life of their communities [46,47,48].	2	2	3	2	3	2	3	17
Pacific	**Children’s Healthy Living:** Childhood obesity among Pacific children was investigated using multiple approaches including (1) program/data inventories and situational analyses; (2) training of professionals and paraprofessionals in obesity prevention; (3) development of a Pacific food, nutrition, and physical activity data management and evaluation system; (4) development and conduct of a community-based environmental intervention to prevent, maintain, or reduce young child overweight and obesity; (5) evaluation of the environmental intervention; and (6) incurring at least one obesity-prevention policy change per jurisdiction [43,49,50].	1	3	3	3	3	3	1	17
Canada	**Healthy Food North:** Attempts to increase consumption of traditional foods (e.g., caribou, fish) and nutrient-dense, store-bought foods low in fat and sugar (e.g., fruits, vegetables); decrease consumption of non-nutrient-dense, high-fat, high-sugar foods (e.g., soda, chips); and increase engagement in moderate and vigorous physical activity while reducing sedentary activity were made including promotional materials, media, and interactive educational activities held in food shops, worksites, and other community venues as well as community-wide events [51].	1	3	3	3	3	3	1	17
	**Healthy Alberta Communities:** Attempts at: (1) reducing prevalence of overweight and chronic disease risk, (2) increasing community capacity to promote health, and (3) informing policy, practice, and research decisions about public health were made through a multitude of physical activity and nutrition intervention approaches that were built upon the premise of community-based participatory research [52,53].	3	3	3	3	3	3	3	21
	**Sustainable Childhood Obesity Prevention through Community Engagement (SCOPE):** A multi-setting, multi-component program designed to enhance community capacity to create and deliver solutions to promote healthy eating, physical activity, and healthy weights among school-aged children was implemented. Underpinned by social ecological theory, community-based and community-driven action facilitated by the best evidence and shared strategies across multiple stakeholders was utilized to promote healthy body weights [54,55,56].	2	3	3	3	3	3	1	18
United Kingdom and Europe	**Healthy towns England:** More than 200 individual interventions, primarily focused on promoting healthy diet and physical activity, were implemented with no reference to community capacity building [57,58].	1	1	1	1	1	1	1	7
	**Neighbourhood Renewal Fund—Obesity Prevention:** A series of interventions aimed at changing nutrition and physical activity behaviors in the local community was implemented without any notable emphasis on capacity building [59].	1	1	2	1	1	1	1	8
	**Healthy Lifestyle Program:** A systematic process—Intervention Mapping (IM)—was applied to plan a school-based obesity-prevention intervention. Several domains of capacity building, including needs assessments, intelligence gathering, and stakeholder consultations, were included in this process [60,61].	1	3	1	3	2	2	1	13
	**Sundhed og Lokalsamfund:** Promotion of healthier lifestyles among Danish children aged 3–8 years and their families was achieved through collaborating with the local educational programs for nurses, school, and kindergarten and by planning for a training of program ambassadors among the local workforces [43,62,63].	2	3	3	2	3	3	1	17
	**Ensemble Prévenons l’Obésité des Enfants:** A coordinated, capacity-building approach aimed at reducing childhood obesity was implemented through the engagement of local environments, childhood settings, and family norms. Specific emphasis on minimizing cultural or societal stigmatization, step-by-step learning, and an experience of healthy lifestyle habits tailored to the needs of all socioeconomic groups was included with all objectives based around (1) political commitment and policy change; (2) securing sufficient fiscal and physical resources; (3) planning, coordinating, and providing the social marketing, communication, and support services for community practitioners and leaders; and (4) using evidence from a wide variety of sources [64,65,66].	1	3	2	2	3	3	3	17

1 = little evidence of explicit reference/strategy; 2 = some evidence; 3 = considerable evidence. Cronbach’s alpha = 0.77.

## Data Availability

Not applicable.
